# 32329 A novel mouse model of Ataxia Telangiectasia for testing small molecule readthrough compounds

**DOI:** 10.1017/cts.2021.430

**Published:** 2021-03-30

**Authors:** Paul Mathews

**Affiliations:** The Lundquist Institute for Biomedical Innovation at Harbor-UCLA Medical Center

## Abstract

ABSTRACT IMPACT: Small molecule readthrough compounds are a promising therapeutic with the potential to overcome nonsense mutations thereby enabling the production of functional ATM protein in patients with Ataxia Telangiectasia OBJECTIVES/GOALS: To generate a novel mouse model of Ataxia-Telangiectasia for testing small molecule readthrough compounds that both expresses a clinically relevant nonsense mutation and recapitulates the major symptoms of the disease, including a progressive loss of motor coordination not previously observed in prior A-T animal models. METHODS/STUDY POPULATION: Using a double-hit strategy to increase genotoxic stress, we generated a novel A-T mouse model that expresses a clinically relevant (c.103C>T) mutation in the Atm gene and a knockout of the functionally related Aptx gene. We then characterized the mouse across multiple domains related to the various symptoms related to the disease. This includes examination of survivability, immunologic function, cancer prevalence, and motor behavior and its associated cerebellar dysfunction and atrophy. Lastly, we tested the ability of small molecule readthrough compounds to enable production of ATM from tissue explants extracted from these ATM deficient mice. RESULTS/ANTICIPATED RESULTS: The double mutant mice display reduced survivability compared to control mice (53% vs. 97%; p<0.0001), dying at a clinically relevant rate of about 30% from thymomas. At postnatal day 400 (P400), only AtmR35X/R35X; Aptx-/- mice, and none of the controls expressing at least one wildtype Atm or Aptx gene develop a motor behavioral deficits that are associate with reduced Purkinje neuron diameter (8.0 ±0.4 µm vs. 9.92 ±0.5; p<0.01) and density (4.3 ±0.2 vs. 6.0 ±0.3 per 100 µm; p<0.05) as well as cerebellar atrophy (cerebellum/forebrain area 0.26 ±0.01 vs. 0.31 ±0.01; p<0.001). ATM deficient mice also display disrupted thymocyte development and metabolic function. When exposed to small molecular readthrough compounds, greater than 50% of the ATM protein is restored. DISCUSSION/SIGNIFICANCE OF FINDINGS: We have created a novel, clinically relevant A-T mouse model that develops a severe ataxia associated with changes in cerebellar function and atrophy as well as demonstrate the potential of SMRT compounds as an A-T therapeutic.

